# Towards organizing health knowledge on community-based health services

**DOI:** 10.1186/s13637-016-0053-x

**Published:** 2016-11-17

**Authors:** Mohammad Akbari, Xia Hu, Liqiang Nie, Tat-Seng Chua

**Affiliations:** 1 School for Integrative Sciences and Engineering, NUS, Singapore, Singapore; 2 School of Computing, NUS, Singapore, Singapore; 3 Department of Computer Science and Engineering, Texas A&M University, College Station, TX, USA

**Keywords:** Consumer health information, Community question-answering, Information organization, Information retrieval

## Abstract

Online community-based health services accumulate a huge amount of unstructured health question answering (QA) records at a continuously increasing pace. The ability to organize these health QA records has been found to be effective for data access. The existing approaches for organizing information are often not applicable to health domain due to its domain nature as characterized by complex relation among entities, large vocabulary gap, and heterogeneity of users. To tackle these challenges, we propose a top-down organization scheme, which can automatically assign the unstructured health-related records into a hierarchy with prior domain knowledge. Besides automatic hierarchy prototype generation, it also enables each data instance to be associated with multiple leaf nodes and profiles each node with terminologies. Based on this scheme, we design a hierarchy-based health information retrieval system. Experiments on a real-world dataset demonstrate the effectiveness of our scheme in organizing health QA into a topic hierarchy and retrieving health QA records from the topic hierarchy.

## Introduction

The emergence of online health information needs has given rise to the establishment of online health services. Broadly speaking, current online health services can be divided into two categories. The first is the professional health provider released sources, such as Yahoo! Health^1^ and WebMD^2^. These sources provide trustworthy and formally-written health information. They are usually well-structured in terms of health topics. The second category is the community-based health services (CHSs), such as HealthTap^3^ and HaoDF^4^. These services allow health seekers to freely post health-oriented questions, and encourage doctors to provide quality answers. Compared to the former sources, CHSs have some intrinsic properties. First, they are crowdsourcing data that are continually growing at a fast pace, and it is thus not practical to organize them manually. Second, they are unstructured and unlabeled in terms of topics, which greatly hinder their retrieval and browsing by user. Third, health seekers and doctors with diverse backgrounds tend to present the same concepts in colloquial style, which leads to a wide vocabulary gap. Together, these pose big challenges for data access and navigation. Recent efforts [1] indicate that organizing the community-contributed data into a hierarchical structure may enhance coarse-grained browsing and fined-grained search.

Several practical systems and research efforts have been dedicated to organizing community-contributed data [1, 2]. Most of these efforts, however, suffer from the following limitations. First, they typically utilized predefined taxonomies in the form of tree structures and expect users or computers to assign data instances into these taxonomies based upon their understanding. However, the available taxonomies in health domain are usually too shallow with broad categorizations. For example, Yahoo! Answer^5^ partitions health data into only nine main categories which are too general to summarize the diverse health information. Some popular topics such as “pregnancy” cannot be directly browsed here, because they do not fall under the predefined fixed category structure. Besides, these fixed taxonomies usually face the problems of being too centralized, conservative, and ambiguous [3]. Moreover, manual assignment by health seeker is probably not applicable since they do not sufficiently understand their health problems. Second, existing efforts enable each data instance to be assigned into only one leaf node of the hierarchy. However, the health records are usually more verbose and complex, and probably convey multiple concerns. They hence should be assigned into more topic-level leaf nodes. Third, topic hierarchy construction approaches in general domain often annotate each node of the hierarchy with frequent occurrence terms or concepts. However, in vertical domain hierarchy construction, such as health domain, labeling nodes with standard terminologies is preferable, since it facilitates data reusability and exchange. Fourth, the existing efforts are unable to adaptively build the skeleton hierarchy. Specifically, the number of children for each given parent node and the number of layers in the entire hierarchy are either extracted from existing external structures or predefined by the so-called domain experts. They are often biased towards specific context or personal perspectives [4].

To overcome these limitations, we propose a top-down scheme that can organize the unstructured health records into a structured hierarchical tree. First, nodes in higher layers of the tree represent abstract topics. These nodes usually do not have clear definition and are thus difficult to be extracted automatically. On the other hand, even though the existing health-related taxonomies are very general, they still capture the high-level structures of the health domain well. We naturally leverage such prior domain knowledge to construct the higher layers of our hierarchy. Second, we propose an expanding approach to perform overlapping partitioning of each node to generate its children. Starting from the higher layer node, we try to obtain a hierarchy of its children. However, without termination criteria, the generated tree will be very huge in which each leaf node may contain only one health record. To address this problem, we propose a shrinkage approach to monitor and infer whether the node is specific enough before expansion. Following the breadth-first tree traversal trajectory, we alternatively employ expansion and shrinkage approaches to inspect each node and generate a proper hierarchy. In addition, all involved nodes are profiled with terminologies selected from the Unified Medical Language System (UMLS) Metathesaurus^6^.

Based on our proposed organization scheme, we develop a hierarchy-based health information retrieval system. Health information search has attracted intensive attentions from industry and academia [5–9]. The effectiveness and efficiency of these efforts, however, are limited due to the inconsistent terms used in health domain and the need for exhaustive search in the entire data corpus. Our application adopts the topic-based matching and performs intelligent pruning of irrelevant branches of the generated hierarchy, and it can boost search performance significantly.

The contributions of our work are threefold:

- To the best of our knowledge, this is the first work on automatic organization of community-contributed health data.
- With prior domain knowledge, we propose a top-down organization scheme where skeleton hierarchy is automatically determined, multiple relations are enabled, and nodes are profiled with terminologies.
- We propose a hierarchy-based health information retrieval system. Extensive evaluations demonstrate its promising performance.

The remainder of this paper is organized as follows. Sections 2 and 3, respectively, detail our organization scheme and our hierarchy-based health information retrieval. Section 4 introduces the representation of QA records and similarity measures used. Experimental results and analysis are presented in Section 5. Section 6 reviews the related work, followed by our conclusion and future work in Section 7.

## Top-down organization scheme

**Fig. 1:**
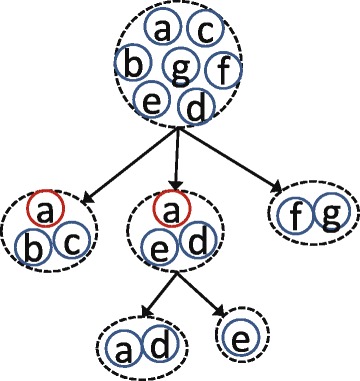
Illustration of loose partition of parent node. *Dashed* and *solid circles* stand for nodes and health QA records, respectively

This paper targets at generating a rooted, directed, and profiled tree *H* from a given data corpus that contains *n* health-related question answering (QA) records $\mathcal {D}=\{x_{1},x_{2},\ldots,x_{n}\}$. Each node $\mathcal {V}$ in *H* is a subset of $\mathcal {D}$, representing a latent topic of semantically similar records. Notably, the root node $\mathcal {V}_{0}$ involves all the records in $\mathcal {D}$. The child nodes loosely partition their parent nodes, where overlapping is allowed. Figure 1 representatively shows the loose partitioning of the given parent node. From this figure, it can be seen that one health QA record can be assigned into two or more sibling nodes.

### Incorporation of domain knowledge

As aforementioned, the current health-related taxonomies are usually very general and shallow. For example, the taxonomies provided by WebMD and Yahoo! Health are almost flat. They typically capture the high-level categorizations and structures of health domain. They are user-oriented categories which model human expectation of abstract categories in health records. On the other hand, automatic extraction of high-level categories of a given corpus is non-trivial, since there are overlaps and inter-correlation between topics especially in health domain. Take the categories of “mental health” and “women’s health” as an example; they are partially overlapped rather than being mutually exclusive and complementary. Regarding the aforementioned discussion, we employed such a domain knowledge to guide the construction of topic hierarchy and ensure that the generated structure is human readable and interpretable. While different kinds of domain knowledge may be available, in this paper, we assume that prior domain knowledge is available as a predefined hierarchy structure. The predefined hierarchy structure may include several layers of nodes labeled with keywords. To facilitate the formalization of our hierarchy generation, in this paper, we utilized a one-level tree structure which includes several child nodes following the root node.

We utilize the categorization of healthexchange^7^ as our initial first layer following the root node. Having a predefined hierarchy structure, we construct a set of classifiers to categorize health QA records in the root node into these categories. To accomplish this task, we first extract a set of exemplar QA pairs to represent the semantic context of each category. To do so, we employ each category’s name as a query and obtain the top 100 relevant QA pairs from HealthTap. To form negative samples, we randomly select 100 negative samples from the other categories. We then trained a SVM classifier using the samples for each category.

### Expanding approach

Through incorporating domain knowledge, we have partitioned the root node into a list of high-level categories which correspond to user expectation of knowledge structure. Each category is viewed as a node in the first layer. This subsection details the expanding approach to further generate a fine-grained hierarchy.

According to our definition, each node $\mathcal {V}$ in the target hierarchy *H* is a set of health QA records. We assume that this collection of health QA records can be explained by a set of unobserved abstract groups, and each group contains a small set of semantically similar health QA records talking about the same health topic. We then naturally shift our expanding task into topic modeling problem. The latent Dirichlet allocation (LDA) model [10] is utilized here, which is a generative model for discovering the unobserved abstract groups that occur in a data collection.

The main challenge in the expanding phase is to determine the proper number of children for each given node. Each child node should represent one aspect of the parent node, and complement to its siblings instead of mutually overlapping. Our proposed expansion approach selects the number of children via a tuning procedure. This procedure seeks for the children number that minimizes the LDA model’s perplexity [10] on a held-out testing data set. It is formulated on a hold-out set with *m* health QA records as

1 $$\begin{array}{*{20}l} \text{perplexity}= \text{exp}\left\{ \frac{\sum_{i=1}^{m} \log p(d_{i})}{\sum_{i=1}^{m} l_{i}}\right\}, \end{array} $$

where *l*_*i*_ is the length of health QA record *d*_*i*_. The lower the perplexity value is, the better is the ability of the corresponding trained model in capturing the text collection.

Based on the proposed expanding approach, nodes in each layer are divided to subtopics where they contain sets of more compact health QA records as compared to their parents. As a byproduct of expansion, we train an optimal LDA model for each node in our generated hierarchy, which is utilized to facilitate health QA records assignment and hierarchy-based retrieval.

### Shrinking approach

Before expanding a given node, we need to estimate how specific the node is, which is the key to automatically determining the depth of the hierarchy and prevents further segmentation of homogeneous nodes. Common approaches predefine a fixed depth and divide the data collection continuously until the depth constraint is satisfied. Approaches of this kind generate balanced trees where all leaves have the same depth. However, they have two limitations. First, the generated hierarchies might be biased towards the experiences of the persons who predefine the depth. Second, the underlying assumption of these approaches is that all sibling nodes have the same complexity and generality, which is not true in health domain. For example, the node talking about “cancer” is more general and should have deeper layers as compared to one that representing “acne.”

We propose a shrinking approach to accomplish this task. Initially, we assume that the given node $\mathcal {V}$ can be further expanded, by dividing it into two child nodes, $\mathcal {A}$ and $\mathcal {B}$. Obviously, $\mathcal {V}$ equals to the union of $\mathcal {A}$ and $\mathcal {B}$, i.e., $\mathcal {V}=\mathcal {A} \cup \mathcal {B}$. We then estimate the average similarity between these two nodes by

2 $$\begin{array}{*{20}l} R(\mathcal{A}, \mathcal{B})=\frac{1}{|\mathcal{A}|\cdot|\mathcal{B}|} \sum\limits_{\mathbf{x}_{i} \in \mathcal{A}, \mathbf{x}_{j} \in \mathcal{B}} S(\mathbf{x}_{i}, \mathbf{x}_{j}), \end{array} $$

where *S*(**x**_*i*_,**x**_*j*_) is their similarity estimation.

Based on the formulation of $R(\mathcal {A}, \mathcal {B})$, we can intuitively have the normalized definitions of inter-node relation and intra-node relation as follows:

3 $$ \left\{\begin{array}{ll} \text{inter}(\mathcal{A}, \mathcal{B})=\frac{R(\mathcal{A}, \mathcal{B})}{R(\mathcal{A}, \mathcal{V})} + \frac{R(\mathcal{A}, \mathcal{B})}{R(\mathcal{B}, \mathcal{V})},\\ \text{intra}(\mathcal{A}, \mathcal{B})=\frac{R(\mathcal{A}, \mathcal{A})}{R(\mathcal{A}, \mathcal{V})} + \frac{R(\mathcal{B}, \mathcal{B})}{R(\mathcal{B}, \mathcal{V})}. \end{array}\right.  $$

The stronger the inter-node relation between $\mathcal {A}$ and $\mathcal {B}$ is, the more indivisible they are. On the other hand, a smaller intra-node relation indicates a more tighter consolidation of $\mathcal {V}$, and hence, it is not necessary to split it further.

In our work, if $\text {inter}(\mathcal {A}, \mathcal {B})$ is larger than our threshold *δ*, we will terminate the expanding phase. The threshold is obtained empirically based on our experiments.

### Health QA record assignment

As aforementioned, health QA records usually involve multiple topics. For example, this question is selected from HealthTap, “what can cause breast cancer to 25 years old married girl within the first 3 months of pregnancy?” It explicitly talks about at least three topics: “breast cancer,” “female health,” and “pregnancy.” Therefore, assigning such records into multiple and complementary child nodes is desired in health domain.



Based on our LDA model, each health QA record in the parent node can be represented as a mixture of all its children topics with different weights, i.e., $p(\mathcal {V}_{i}|\mathbf {x})$, denoting the probability of a health QA record **x** associated to a child node $\mathcal {V}_{i}$. Some child nodes with larger probabilities capture the principle components of the given health QA record, while others play supporting roles. However, there is not an indisputable approach to determine how many nodes should be selected for the assignment. If we choose too many child nodes, we may bring in noise for those nodes that are not the principle topics of the given health QA record. If we choose too few, we lose relevant category information of the given health QA record. As a rule of thumb, we should select only the leading interpretable child nodes.

**Fig. 2:**
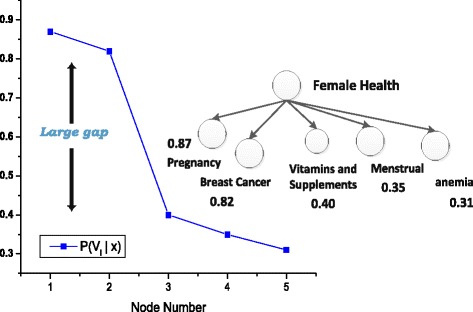
Illustration of leading nodes selection

An important observation reveals that the leading child nodes make significantly larger impact than other supporting child nodes. As Fig. 2 shows, there is a large gap between the impact of leading child nodes, i.e., *v*_1_ and *v*_2_, and that of the supporting child nodes, i.e., *v*_3_, *v*_4_, and *v*_5_. This gap shows that the given QA record is highly relevant to the first two child nodes while it is less relevant to the last three child nodes. Hence, we assign the current health QA record, i.e., **x**, into just highly relevant child nodes, i.e., *v*_1_ and *v*_2_ in our example. Based on this observation, the number of leading child nodes can be heuristically selected according to Algorithm 1. The algorithm first calculates the difference between two adjacent values in the ranked list of child nodes (line 2). It then finds the maximum difference to compute the number of leading nodes for current health QA records (line 3). The complexity of this algorithm is *O*(*nlogn*). Similar approach was utilized to determine the leading roles from movies [11].

### Node profiling with terminologies

Our LDA-based top-down scheme automatically extracts child nodes in the form of multinomial distributions of words from the parent node. In general, it is very difficult for users to understand a child node only based on the multinomial distribution of words, especially when they are not familiar with the context. Consequently, we need to generate meaningful labels for each node to ease understanding. In this section, we propose an approach for profiling nodes of the constructed hierarchy with medical terminology.

Early literatures [10, 12, 13] on topic labeling generally either select the top statistical terms in the distribution as primitive labels or generate labels manually in a subjective manner. These approaches, however, are not applicable to CHSs due to the following reasons. First, frequent terms might not be medical concepts, such as “desktop.” Second, terms are less descriptive than phrase-based concepts. Third, manual generation is time-consuming and error-prone. In addition, terms are not standardized and inconsistent. Therefore, it is essential to automatically profile nodes with phrase-based standard terminologies.

Given one node, we initially assign part-of-speech tags to each word for all the health QA records associated with this node^8^. We then extract the noun phrases where their tag sequences match a fixed pattern,

4 $$\begin{array}{*{20}l} &(\text{Adjective} | \text{Noun})^{*} (\text{Noun}\quad \text{Preposition})\\  & ? (\text{Adjective} | \text{Noun})^{*} \text{Noun}. \end{array} $$

A sequence matching this pattern ensures a noun phrase, such as the phrase “ineffective treatment of terminal lung cancer.” We do basic post processing to link the variants of terms together, such as singularizing all plural variants.

We select the top *k* frequent noun phrases $\mathcal {C}=(c_{1},c_{2},\ldots,c_{k})$ and normalize them into authenticated terminologies in ULMS via a voting method. More specifically, we first use MetaMap tool^9^ to map each phrase into the ULMS terminology. It is worth highlighting that some distinct noun phrases may be mapped to the same terminology. For example, “painful neck” and “neck ache” are both normalized to “neck pain.” We next use a voting strategy to rank terminology candidates $\mathcal {T}=(t_{1},t_{2},\ldots t_{m})$ and produce the final labels by selecting the top ones,

5 $$\begin{array}{*{20}l} \text{score}(t_{i}) = \sum\limits_{j=1}^{k} \text{vote}(c_{j}, t_{i}), \end{array} $$

where vote(*c*_*j*_,*t*_*i*_) is a binary form definition

6 $$ \text{vote}(c_{j}, t_{i})= \left\{\begin{array}{ll} 1& \text{if \(t_{i}\) is terminology of \(c_{j}\)}\\ 0& \text{otherwise} \end{array},\right.  $$

where Eq. (5) aggregates all the votes for each terminology phrase and Eq. (6) increases the score of a terminology if it can be inferred from a noun phrase.

A ranking list of terminologies for each node can be generated and the top ones are truncated as labels. The above voting approach preserves two characteristics. First, it assigns higher score to medical terminologies which are relevant to frequent occurring noun phrases in the cluster. Second, by inferring medical terminologies using MetaMap tool, we indeed normalize noun phrases into a standard medical terminology, i.e., UMLS.

## Hierarchy-based retrieval

Reported by a national survey, which was conducted by the Pew Research Center^10^, retrieval is the main mode of acquiring health information by users. Keyword-based indexing and matching is the prevailing method of retrieval. However, it is not sufficient for healthcare domain because of the complex, inconsistent and ambiguous terms used by users. In fact, the same questions may be described in substantially different ways by two individual health seekers, even by the well-trained doctors. For example, the query “I want to get pregnant what is the first thing I should do in diet and supplementary term?” and the archived health QA record “what are the best vitamins for a woman who decides to have a child soon?” are too semantically similar and both talking about mothers’ worries about pregnancy. However, they are not very syntactically similar to be matched.

To boost the search performance, we propose a hierarchy-based retrieval application. It first deems the given query as a health QA record and performs health QA record assignment to the offline generated hierarchy. This is done by routing the given query from root level down to appropriate leaves of the tree. Obviously, this process plays an essential role in pruning the search space via routing the given query to the relevant branches. Meanwhile, the health QA record assignment actually employs the topic-based representation to semantically match the query to the relevant branches, which naturally tackles many of the limitations associated with term-based matching.

For a given query, a small set of leaf nodes are located. However, the health QA records within these selected leaf nodes are still large that will easily overwhelm the health seekers. Therefore, ranking these health QA records and returning the top ones to the health seekers will enrich the users’ search experiences. The existing ranking approaches generally fall into two classes [14, 15]. One is pseudo relevance feedback based [16–18], which treats a significant fraction of the top documents as pseudo-positive examples and collects some bottom documents as pseudo-negative examples. They then either learn a classifier or cluster the documents to perform ranking. The other class is graph based [19–22] that propagates the initial ranking information over the whole graph until convergence. Inspired by [19], we adopt the graph-based random walk ranking method, which is formulated based on two assumptions:

1. The relevance probability function is continuous and smooth in semantic space. This means that the relevance probabilities of semantically similar health QA records should be close.
2. The final relevance probabilities should be close to the initialized ones for each health QA record.

We construct a graph where the vertices are health QA records and the edges reflect their pairwise similarities. We first introduce some notations. We use **W** to denote the initialed similarity matrix and *W*_*ij*_, its (*i,j*)th element, indicates the similarity of **x**_*i*_ and **x**_*j*_, estimated using Eq. (13). Let *d*_*ii*_ denote the sum of the *i*th row of **W**, i.e., $d_{ii}=\sum _{j}W_{ij}$. Then, the graph-based learning approach can be written as

7 $$\begin{array}{*{20}l} \min\limits_{\mathbf{y}}\frac{1}{2}\sum\limits_{i,j}W_{ij}\left(\frac{y_{i}}{d_{ii}} - \frac{y_{j}}{d_{jj}}\right)^{2}+\lambda\sum\limits_{i}\frac{1}{d_{ii}}(y_{i}-\bar{y}_{i})^{2}, \end{array} $$

where *λ* is a weighting parameter and *y*_*i*_ is the relevance probability of **x**_*i*_ that we want to estimate. $\bar {y}_{i}$ is the initialized relevant score estimated by Eq. (13). We can see that the smoothness assumption is enforced in the first term of the above equation, which enforces the relevance probabilities of semantically similar health QA records to be close. The second term reflects the second assumption, i.e., the probabilities we estimate should be close to the ranking-based probabilities.

We use **D** to denote a diagonal matrix, with *d*_*ii*_ to be its (*i,i*)th element; and let **g** denote $\left [\frac {y_{1}}{d_{11}},\frac {y_{2}}{d_{22}},\ldots,\frac {y_{n}}{d_{nn}}\right ]^{T}$. Thus, Eq. (7) can be rewritten as,

8 $$\begin{array}{*{20}l} \min_{\mathbf{g}} \mathbf{g}^{T}(\mathbf{D}-\mathbf{W})\mathbf{g} + \lambda (\mathbf{g}-\mathbf{D}^{-1}\bar{\mathbf{y}})^{T}\mathbf{D}(\mathbf{g}-\mathbf{D}^{-1}\bar{\mathbf{y}}). \end{array} $$

It can be derived that

9 $$\begin{array}{*{20}l} \mathbf{y}=\frac{1}{1+\lambda}\mathbf{WD}^{-1}\mathbf{y}+\frac{\lambda}{1+\lambda}\bar{\mathbf{y}}. \end{array} $$

We can iterate the above equation and the convergence can be proven. With graph-based random walk ranking, we return an ordered list of health QA records to health seekers.

## Features and similarity estimation

To represent QA records, we extract lexical, syntactic, and semantic features.

***Weighted term kernel Φ***_***1***_: Medical concepts usually convey more informative signals than others. It is reasonable to assign greater weights to these concepts. We propose a weighted bag-of-word approach to lexically represent health QA content. Specifically, medical concepts falling into certain UMLS semantic groups will be weighted twice [23]. These groups include disease or syndrome, body part organ or organ component, sign or symptoms, and neoplasm. These groups are chosen since they cover most of the medical concepts and the medical concepts within them are discriminative. Cosine similarity is then employed to calculate the lexical similarity between two QA records.

***Syntactic tree kernel Φ***_***2***_: The tree kernel function is one of the most effective ways to represent the syntactic structure of a sentence [24]. The tree kernel was designed based on the idea of counting the number of tree fragments that are common to both parsing trees, and defined as

10 $$\begin{array}{*{20}l} STKN(T_{i}, T_{j}) = \sum\limits_{n_{i} \in T_{i}} \sum\limits_{n_{j} \in T_{j}} C(n_{i}, n_{j}), \end{array} $$

where *n*_*i*_ and *n*_*j*_ are sets of nodes in two syntactic trees *T*_1_ and *T*_2_, and *C*(*n*_*i*_,*n*_*j*_) equals to the number of matched sub-trees rooted in nodes *n*_*i*_ and *n*_*j*_, respectively. STKN is originally designed to measure the similarity between two sentences. However, health QA records usually includes multiple sentences. We thus generalize it to *Φ*_2_ as

11 $$\begin{array}{*{20}l} \Phi_{2}= \frac{\sum_{s_{i} \in d_{1}} \sum_{s_{j} \in d_{2}} STKN(T(s_{i}), T(s_{j}))}{\vert d_{1}\vert \vert d_{2} \vert}, \end{array} $$

where *s*_*i*_ and *s*_*j*_ are sentences from *d*_1_ and *d*_2_, respectively. In this way, we moderate the effects of the length of health QA records.

***Latent topic kernel Φ***_***3***_: We explore the LDA-based high-level representation. For a collection of health QA records, LDA assigns semantically interrelated health concepts into the same latent group, which can be used to describe the underlying semantic structures of health data in the context of a hierarchical topic. In our work, each group is deemed as one feature dimension. Hence, for a given health QA record, it can be represented as a mixture of latent groups. The feature dimensions are determined via perplexity score.

Traditionally, the Kullback-Leibler divergence (KL-divergence) is used to compute the similarity between two topic distributions. However, KL-divergence is asymmetry per se, which makes it difficult to be used as a similarity metric. To address the asymmetry of KL-divergence, we utilize the Jensen-Shannon divergence scores as follows:

12 $$\begin{array}{*{20}l} \Phi_{3}= 0.5KL(p_{1} \Vert q)+0.5 KL(p_{2} \Vert q), \end{array} $$

where *KL*(.∥.) denotes KL-divergence score and *q*=0.5*p*_1_+0.5*p*_2_ [25].

To estimate the similarity between two QA records, we linearly fuse these three aspects,

13 $$\begin{array}{*{20}l}  & \Phi = \sum\limits_{i=1}^{3} \beta_{i} \Phi_{i}, \end{array} $$

where *β*_*i*_ sums up to 1, and each of them is greater than 0. We conduct a grid search with step size 0.05 within [0,1] to tune *β*_1_ and *β*_2_ while *β*_3_=1−*β*_1_−*β*_2_. The values that achieved the best results are selected.

## Experiments

### Experimental settings

We collected approximately 109 thousand questions from HealthTap. For each question, we also collected its answers and tags, which are provided by doctors. Compared to normal documents, health questions are short and consist of only a few sentences. They thus do not provide sufficient word co-occurrences or shared contexts for effective similarity measurement. To compensate for this problem, we utilized corresponding answers and tags to contextualize the question parts. Note that for the hierarchy-based search, the newly incoming query contains only the question part.

For the subsequent subjective evaluations, we invited three volunteers who majored in medicine. They were trained with short tutorials and a set of typical examples before their labeling. A majority voting scheme among the volunteers was adopted to alleviate the problem of ambiguity.

### On hierarchy generation

Currently, there are no widely accepted metrics to measure how well the generated hierarchy can explain the given data corpus. In our work, we propose objective and subjective approaches. We compare among three schemes: our scheme without domain knowledge, our scheme with domain knowledge, and hierarchical LDA (hLDA). The hLDA model [26] represents the distribution of topics within documents by organizing the topics into a tree. For hLDA, we assigned each health QA record into one child node based on the generative probability. We profiled each node with terminologies via mapping the top terms in each node to terminologies.

#### On objective hierarchy evaluation

We objectively evaluate the generated hierarchies from local and global angles. Both of these two evaluation approaches view external standard medical knowledge structure as golden hierarchies. In our work, we chose medical subject headings^11^ (MeSH) as ground truth. It is a national library of medicines controlled vocabulary thesaurus. It consists of sets of terms naming descriptors in a hierarchical structure that permits searching at various levels of specificity. MeSH descriptors are arranged in both an alphabetic and a hierarchical structure. At the most general level of the hierarchical structure are very broad headings such as “anatomy” or “mental disorders.” More specific headings are found at the narrower levels of the 12-level hierarchy, such as “ankle” and “conduct disorder.” There are 27,149 descriptors in *2014* MeSH.

For the local evaluation, we estimated the proportion of correct parent-child relations between labeled terminologies. We first formed a collection of relation tuples (parent, child) from the profiled hierarchies. We then inspected the correctness of each tuple in MeSH.

However, there exist some parent-child relations in our generated hierarchies which cannot be identified exactly in MeSH. For example, terminology *t*_*i*_ may be a grandchild of *t*_*j*_ in MeSH, while it is a child of *t*_*j*_ in the generated hierarchies. Therefore, local metrics are unable to comprehensively reflect the hierarchy cohesiveness. That motivates a global measure to estimate the cohesiveness,

14 $$\begin{array}{*{20}l} \text{cohesivenss} =\frac{1}{M \cdot N} \sum\limits_{i=1}^{M} \sum\limits_{j=1}^{N} R(t_{i}, t_{j}), \end{array} $$

where *t*_*i*_ is the terminology in the parent node of the generated hierarchy, while *t*_*j*_ is the terminology in the adjacent child node. *R*(*t*_*i*_,*t*_*j*_) is calculated based on MeSH,

15 $$ R(t_{i}, t_{j})= \left\{\begin{array}{ll} \frac{1}{2^{p}}& \text{if ancestor-child relations}\\ 0& \text{otherwise} \end{array},\right.  $$

where *p* is the length of ancestor-child path between terminology *t*_*i*_ and *t*_*j*_.

The local and global evaluation results are presented in Table 1. It can be seen that our approaches extract much more relations between concepts from corpus. Moreover, our approaches outperform the hLDA in terms of local and global evaluations. The low cohesiveness values are caused by the fact that some parent-child terminologies are not represented in MeSH in the ancestral series. Finally, even though we use a very basic domain knowledge, it boosts the performance of the hierarchy generation, which validates the importance of domain knowledge for organizing medical data.

**Table 1:** Local and global evaluation results of the generated hierarchies

Approaches	Total tuples	Correct tuples	Accuracy (%)	Cohesiveness
hLDA	74	17	22.97	1.2 ×10^−4^
Ours without domain knowledge	398	158	39.70	2.0 ×10^−4^
Ours with domain knowledge	326	134	41.10	3.1 ×10^−4^

#### On subjective hierarchy evaluation

As a complementary evaluation approach, we subjectively validated the generated hierarchies. We asked the volunteers to not only focus on the high-level parent-child relations in terms of labeled terminologies, but also the fine-grained context of the generated hierarchy. Because the hierarchies are very large, we first segmented each hierarchy into several tree fragments based on three conditions:

- Each fragment contains at most two levels.
- At most four siblings are allowed.
- Fifty records were randomly sampled from each selected node to represent its context.

The volunteers were required to go through all the health QA records in each fragment, which help them to grasp the contexts. After that, they were asked to annotate each fragment with ratings of “very satisfied,” “satisfied,” and “not satisfied.” The results are presented in Table 2. As can be seen, our proposed schemes significantly outperform hLDA. Meanwhile, the hierarchy generated with domain knowledge can further reduce the “not satisfied” cases. We also evaluated the inter-volunteer agreement with the Kappa method [27]. The overall agreement value is 85.99*%*, while the fixed-marginal Kappa and free-marginal Kappa values are 0.7736 and 0.7899, respectively. They demonstrate that there are sufficient inter-volunteer agreements.

**Table 2:** Subjective evaluation of generated hierarchies

Approaches	# of fragments	Very satisfied	Satisfied	Not satisfied
hLDA	50	16	7	27
Ours without domain knowledge	50	34	10	6
Ours with domain knowledge	50	32	14	4

#### On health QA records assignment

Our scheme enables each health QA record to be assigned into multiple siblings. According to our statistics, on average each record is categorized into 1.7 child nodes. We aim to evaluate the precision and recall of our assignment approach. Precision equals to the number of correctly assigned child nodes over all assigned child nodes, while recall measures the fraction of principle topics of the given health QA record that are captured by the assigned child nodes. As aforementioned, for hLDA, each health QA record in the parent node was assigned into only one child node, which serves as a baseline to see how well our assignment approach performs.

Specifically, we randomly selected 20 nodes and their child nodes from each of the three hierarchies. For each node, we randomly sampled 10 health QA records. Three volunteers were first asked to go through each node and their child nodes to understand what subtopic each child node stands for. In fact, this stage provides cues to the volunteers to which child nodes the given health QA record should be assigned. Suppose the volunteer thinks that the given health QA record should be assigned into *v* child nodes, while it was only correctly assigned into *u*, then the recall for this health QA record is *u*/*v*. Average recall over three volunteers was calculated for each health QA record. Naturally, we also obtained the assigning precision for each health QA record. Table 3 presents the results. It can be seen that our schemes show superiority over hLDA. Our scheme with domain knowledge achieves promising performance in terms of recall.

**Table 3:** Subjective evaluation of assignments of health QA records into hierarchies

Approaches	# of selected nodes	# of Sampled records	Recall (%)	Precision (%)
hLDA	20	200	48.2	76.5
Ours without domain knowledge	20	200	61.7	82.56
Ours with domain knowledge	20	200	65.86	82.33

#### On node profiling with terminologies

It is well known that for the labeling task, precision is usually more important than recall. We thus adopted two metrics that are able to characterize precision from different aspects. The first one is average *S* *@* *K* over all testing nodes, which measures the probability of finding a relevant terminology among the top K recommended candidate terms. To be specific, for each testing node, *S* *@* *K* is assigned to 1 if a relevant terminology is positioned in the top *K* terms and 0 otherwise. The second one is average *P* *@* *K* that measures the proportion of recommended terminologies that are relevant. It is formulated as $P@K =\frac {\mid \mathcal {C} \cap \mathcal {R} \mid }{\mid \mathcal {C} \mid }$, where $\mathcal {C}$ is a set of top *K* terminologies and $\mathcal {R}$ is the manually labeled positive ones. The volunteers were required to label only top five suggested terminologies for each node, and they were labeled either as “positive” or “negative.”

Table 4 illustrates the results in terms of *S* *@* *K* and *P* *@* *K*. It can be seen that our methods consistently outperform hLDA in both *S* *@* *K* and *P* *@* *K*. This may be caused by the use of frequent terms in hLDA that are not medical terms.

**Table 4:** The evaluation results of node profiling with terminologies in terms of *S* *@* *K* and *p* *@* *K*

	S@1 (%)	S@3 (%)	S@5 (%)	P@1 (%)	P@3 (%)	P@5 (%)
hLDA	50	72	100	50	48.33	45
Ours without domain knowledge	52.5	80	100	52.5	50.83	46.5
Ours with domain knowledge	57.5	87.5	100	57.5	49.17	48

### On hierarchy-based retrieval

We comparatively evaluate the following unsupervised reranking methods:

- **KB**: term-based matching was implemented based on Apache Lucene^12^ via indexing all health QA records in our data corpus.
- **PRF**: pseudo-relevance feedback [16].
- **R_noDK**: retrieval based on our scheme without domain knowledge.
- **R_DK**: retrieval based on our scheme with domain knowledge.

To obtain the relevance ground truth of returned health QA record, we conducted a manual labeling procedure. Each health QA record was labeled by three volunteers to be very relevant (score 2), relevant (score 1), or irrelevant (score 0) with respect to the given query. We adopted *NDCG* *@* *n* as our metric [28].

**Fig. 3:**
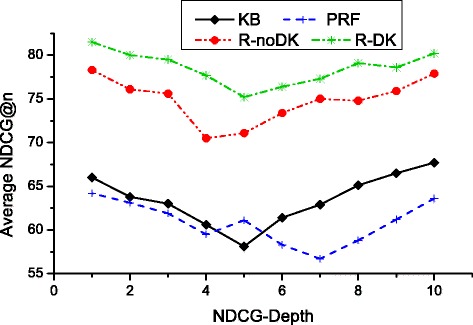
Performance comparison among search algorithms in terms of NDCG@N

We randomly sampled 50 questions as queries. Figure 3 illustrates the experimental results with various *NDCG* depths. It can be observed that our proposed hierarchy-based retrieval approaches consistently outperform the other prevailing techniques. The possible reason may be the different search space. **KB** and **PRF** search over the entire data corpus, while ours route the given query to relevant leaf nodes that ensures the relevant search space in semantic topic level. The following graph-based random walk reranking further improves the precision. In addition, the **R_DK** approach performs better than **R_noDK**, because our scheme without domain knowledge is unable to precisely partition high-level groups.

## Related work

Related literatures on organizing user-generated contents can roughly be classified into three categories: pattern-based, statistical, and folksonomy-based approaches.

The pattern-based approaches utilize predefined linguistic rules to identify concepts and their inter-relations, such as “is-a” and “whole-part.” For example, Li et al. [29] defined a subsumption relation to extract ontological relations between complex concepts from text segments. Beyond hierarchy generation on individual data source, the effort in [2] concentrated on organizing information resources into a topic hierarchy from multiple independent sources.

Statistical approaches either use hierarchical clustering methods or build a model to generate the hierarchy. For instance, Ming et al. [1] clustered web knowledge based on a predefined prototype hierarchy. Cimiano and Staab [30] constructed a hierarchy using agglomerative clustering and a hypernym oracle. Another example, Wang et al. [31] used generative model to cluster concepts for organizing information sources.

Folksonomy-based approaches attempt to generate hierarchies in lights of the collaborative annotated tags. Tang et al. [32] presented an ontology learning method using generative probabilistic model. Tsui et al. [33] used heuristic rules and a concept-relation acquisition schema to convert folksonomies to taxonomy. Song et al. [34] proposed a hierarchical tag visualization approach based on greedy algorithm. They then iteratively selected an optimal tag from the ranking list and inserted it into the tree following the minimum-evolution criteria.

However, most of these approaches are not suitable for CHSs due to the following issues. First, they usually allow each data instance to be assigned into only one leaf node. While each record in health domain usually covers more than one concern. Second, they label each node by a set of frequent concepts and terms instead of standard terminologies, which is not feasible for inter-system operations. Most importantly, the existing efforts do not consider flexible number of sub topics and layers for topic hierarchies.

## Conclusions

This paper presented a novel top-down hierarchy generation scheme that is able to automatically organize the community-contributed health data with prior domain knowledge. Each node in the generated hierarchy was labeled with terminologies. Meanwhile, each health record can be categorized into more than one leaf nodes. Based on the generated hierarchy, a search function was designed and implemented to boost health information retrieval performance.

Our future work will focus on query-aware hierarchy generation. Specifically, given a natural language query, we will return a comprehensive hierarchy that covers various aspects expected by the query.

## Endnotes

^1^ http://health.yahoo.net

^2^ http://www.webmd.com

^3^ https://www.healthtap.com

^4^ http://www.haodf.com

^5^ http://sg.answers.yahoo.com

^6^ http://www.nlm.nih.gov/research/umls/

^7^ http://www.healthxchange.com.sg

^8^ http://nlp.stanford.edu/software/tagger.shtml

^9^ http://metamap.nlm.nih.gov/

^10^ http://pewinternet.org/Reports/2013/Health-online.aspx

^11^ http://www.nlm.nih.gov/mesh/

^12^ http://lucene.apache.org
